# Integrated profiling identifies ITGB3BP as prognostic biomarker for hepatocellular carcinoma

**DOI:** 10.17305/bjbms.2021.5690

**Published:** 2021-12

**Authors:** Qiuli Liang, Chao Tan, Feifei Xiao, Fuqiang Yin, Meiliang Liu, Lei Lei, Liuyu Wu, Yu Yang, Hui Juan Jennifer Tan, Shun Liu, Xiaoyun Zeng

**Affiliations:** 1 Department of Epidemiology and Statistics, School of Public Health, Guangxi Medical University, Nanning, China; 2 Department of Epidemiology and Statistics, School of Public Health, Guilin Medical University, Guilin, China; 3 Department of Epidemiology and Biostatistics, University of South Carolina, Columbia, SC, United States; 4 Life Sciences Institute, Guangxi Medical University, Nanning, China; 5 School of Life Sciences & Chemical Technology, Ngee Ann Polytechnic, Singapore; 6 Key Laboratory of High-Incidence-Tumor Prevention and Treatment, Guangxi Medical University, Ministry of Education, Nanning, China; 7 Hospital Infection Management Department, Liuzhou Workers’ hospital, Liuzhou, China

**Keywords:** Hepatocellular carcinoma, biomarker, hub genes, prognosis, ITGB3BP

## Abstract

Hepatocellular carcinoma (HCC) is a highly malignant tumor. In this study, we sought to identify a novel biomarker for HCC by analyzing transcriptome and clinical data. The R software was used to analyze the differentially expressed genes (DEGs) in the datasets GSE74656 and GSE84598 downloaded from the Gene Expression Omnibus database, followed by a functional annotation. A total of 138 shared DEGs were screened from two datasets. They were mainly enriched in the “Metabolic pathways” pathway (Padj = 8.21E-08) and involved in the carboxylic acid metabolic process (Padj = 0.0004). The top 10 hub genes were found by protein-protein interaction analysis and were upregulated in HCC tissues compared to normal tissues in The Cancer Genome Atlas database. Survival analysis distinguished 8 hub genes CENPE, SPDL1, Hyaluronan-mediated motility receptor, Rac GTPase activating protein 1, Thyroid hormone receptor interactor 13, cytoskeleton-associated protein (CKAP) 2, CKAP5, and Integrin subunit beta 3 binding protein (ITGB3BP) were considered as prognostic hub genes. Multivariate cox regression analysis indicated that all the prognostic hub genes were independent prognostic factors for HCC. Furthermore, the receiver operating characteristic curve revealed that the 8-hub genes model had better prediction performance for overall survival compared to the T stage (p = 0.008) and significantly improved the prediction value of the T stage (p = 0.002). The Human Protein Atlas showed that the protein expression of ITGB3BP was upregulated in HCC, so the expression of ITGB3BP was further verified in our cohort. The results showed that ITGB3BP was upregulated in HCC tissues and was significantly associated with lymph node metastasis.

## INTRODUCTION

Hepatocellular carcinoma (HCC) is a major global public health problem. It has become one of the six most common malignant tumors in the world and is the third cause of cancer-related deaths [1]. Currently, the incidence and mortality of HCC are still on the rise, with about 841,000 new cases of HCC and 782,000 deaths annually worldwide [2]. Due to the occult onset of liver cancer, more than 60% of patients are diagnosed with intermediate-stage or advanced-stage HCC [3] and have a poor prognosis, with the median survival of advanced patients being <1 year [4]. Furthermore, HCC has a high potential for recurrence and metastasis. Invasion and metastasis, intrahepatic metastasis, tumor thrombus formation, and satellite nodules are important factors for HCC recurrence and poor outcome [5], leading to low survival rate for HCC patients. Moreover, due to the limited choice of treatment options, the survival of HCC patients after treatment is still not optimistic [3]. Therefore, the search for biomarkers related to the prognosis of HCC is of great significance in predicting the prognosis of HCC patients, guiding clinical treatments, and improving the prognosis of HCC patients.

Over the decades, an increasing number of genes have been demonstrated to be involved in the development of HCC and may serve as prognostic biomarkers for HCC. PLCE1, for example, an oncogene for HCC, affects the prognosis of patients by influencing the cell cycle, proliferation, migration, and invasion of HCC [6]. High expression of CLEC4M is associated with microvascular invasion, increased tumor volume, loss of tumor encapsulation, decreased tumor differentiation, predicted poor relapse-free survival, and overall survival (OS) [7]. BICD1 is highly expressed in HCC and is positively correlated with malignant clinical characteristics. As its downregulation inhibits the biological function of HCC, it may become a potential prognostic marker and therapeutic target for HCC [8]. Moreover, Sp2 may play a role in promoting cancer by regulating the expression of the TRIB3 protein and may be one of the factors affecting the prognosis of HCC [9]. These findings suggest that dysregulated genes expression may play a crucial role in the outcome of HCC. The search for prognostic markers of HCC can effectively guide the treatment of HCC. Although the abnormally expressed genes in HCC have been extensively studied, the effectiveness of prognostic predictions and therapeutic strategies for this deadly disease remains limited. Therefore, the role of differentially expressed genes (DEGs) in HCC remains to be further studied.

Bioinformatics research has been widely used and plays an important role in cancer research [10]. Public databases such as Gene Expression Omnibus (GEO) database and The Cancer Genome Atlas (TCGA) database contain large amounts of sequencing data of tumor tissues and the corresponding normal tissues of a variety of human cancers. It provides an opportunity for us to investigate multiple diagnostic or prognostic biomarkers at once [11], allowing new biomarkers for HCC to be found [12,13]. In the present study, a total of 138 shared differentially expressed genes (SDEGs) were identified in two independent microarray datasets (GSE74656 and GSE84598) which includes tumor tissue (TT), tumor invasive tissue (TIT), and normal tissue (NT) gene expression profiling. CENPE, SPDL1, Hyaluronan-mediated motility receptor (HMMR), Rac GTPase activating protein 1 (RACGAP1), Thyroid hormone receptor interactor 13 (TRIP13), cytoskeleton-associated protein 2 (CKAP2), CKAP5, and Integrin subunit beta 3 binding protein (ITGB3BP) were identified as prognostic biomarkers and therapeutic targets for HCC. Further analysis demonstrated that ITGB3BP was a promising prognostic biomarker for HCC.

## MATERIALS AND METHODS

### Data preparation

We used cancer, tumor, carcinoma, neoplasm, HCC, and liver as retrieval keywords to retrieve HCC related GEO datasets in the GEO database (http://www.ncbi.nlm.nih.gov/geo/) (up to October 25, 2019). Datasets containing gene expression profiling in TT, TIT, and NT were used for further analysis. Finally, two independent datasets GSE74656 (including 5 groups of TTs, TITs, and NTs) and GSE84598 (including 22 groups of TTs, TITs, and NTs) were downloaded from GEO database. A total of 19,097 genes from GSE74656 and 31,023 genes from GSE84598 were included for analysis. The RNA sequencing data and clinical information of 371 HCC TT and 50 NTs were obtained from the TCGA database (https://cancergenome.nih.gov/) (up to October 30, 2018). The RNA sequencing data were standardized at log_2_ (TPM).

### Analysis of DEGs

The R software “limma” package was employed to detect the differentially expressed mRNAs between TT, TIT, and NT in GSE74656 and GSE84598. To identify DEGs, the thresholds were set at the absolute value of log2 fold change >1.0 as well as adjusted *p* < 0.05. Then, the DEGs common to both datasets were identified for subsequent analysis.

### Protein-protein interaction (PPI) analysis and functional annotation

The PPI network was performed with the Search Tool for Retrieval of Interacting Genes (STRING) (https://string-db.org/cgi/input.pl) and visualized with Cytoscape software. The Cytohubba module in Cytoscape software is an important tool for further gene screening, selecting the important genes, called hub genes, using the algorithm’s calculation, and analysis of the network structure and the weighted connections between nodes. Through this module, we screened the top ten most significant hub genes according to the score ranked by MCC method for further analysis. An online tool KOBAS 3.0 (http://kobas.cbi.pku.edu.cn/kobas3) was used for Gene Ontology (GO) analysis and Kyoto Encyclopedia of Genes and Genomes (KEGG) pathway enrichment to explore gene function. The GO annotation had three parts: Cellular component, biological process, and molecular function.

### HCC samples

A total of 69 paired of HCC tissues and para-carcinoma tissues were obtained from the Affiliated Cancer Hospital of Guangxi Medical University in China. Informed consent was obtained from each patient before participating in the study. The present study was approved by the Ethics Committee of Guangxi Medical University (20170228-3).

### Quantitative reverse transcription polymerase chain reaction (qRT-PCR)

Total RNA was extracted from tissues using TRIzol® reagent (Invitrogen; Thermo Fisher Scientific, USA) and was reverse transcribed using the TaKaRa two-step method (Takara Bio, Dalian, China) according to the manufacturer’s protocols. ITGB3BP and GAPDH were amplified using the TB GreenTM Premix Ex TaqTM II Kit (Takara) in the StepOnePlus real-time PCR system (Applied Biosystems) following the manufacturer’s instructions. The relative expression of ITGB3BP was calculated using the 2^−DDCt^ method and normalized to GAPDH. The sequences of ITGB3BP and GAPDH primers are as follows: ITDB3BP-F (5’-3’): ACT TCC TCA CAA AGC ATC ACG, ITDB3BP-R (5’-3’): GGC AGA TGC AGA AGT TGG TG; GAPDH-F (5’-3’): AGC CAC ATC GCT CAG ACA C, GAPDH-R (5’-3’): GCC CAA TAC GAC CAA ATC C.

### Ethical statement

The studies involving human participants were reviewed and approved by The Ethics Committee of Guangxi Medical University (20170228-3). The participants provided their written informed consent to participate in this study.

### Statistical analysis

To discover the prognostic hub genes, the GraphPad Prism 5 software was used to analyze the association between gene expression and survival of 371 HCC patients in the downloaded TCGA data and formed survival curves. The medians of gene expression (CENPE: 0.96; CKAP2: 2.67; CKAP5: 4.31; HMMR: 2.48; ITGB3BP: 2.15; RACGAP1: 2.97; SMC2: 2.46; SPDL1: 2.01; TRIP13: 1.82; ZWILCH: 2.24) were set as the cutoff values and the patients were divided into low-expression and high-expression groups. The Kaplan–Meier method was used for survival analysis, and log-rank test *p* < 0.05 indicated that the survival curves were statistically significant.

A total of 50 pairs of HCC TT and NT in TCGA database were used to validate differentially expressed hub genes using paired t-tests. Chi-square test was used to test the experimental data of Immunohistochemical assay (IHC) from The Human Protein Atlas (https://www.proteinatlas.org/). Receiver Operating Characteristic (ROC) curve was constructed to evaluate the performance in diagnosis and prognosis prediction by different models in HCC. Delong’ test was performed to compare the area under the curves (AUCs) of different models [14]. Student’s t-test or the Chi-square test was used to analyze the association between the expression of prognostic hub genes and clinicopathological features of HCC patients. Then, multivariate cox regression analysis adjustment for clinicopathological characteristics including age, gender, histology grade, pathological stages, T stages, Alpha-fetoprotein (AFP) level (take 200 ng/ml as the boundary value), and vascular invasion of patients was done to figure out whether any of prognostic hub genes could be considered as independent prognostic factors for HCC. All two-tailed *p* < 0.05 were considered statistically significant.

## RESULTS

### DEGs identification

**FIGURE 1:**
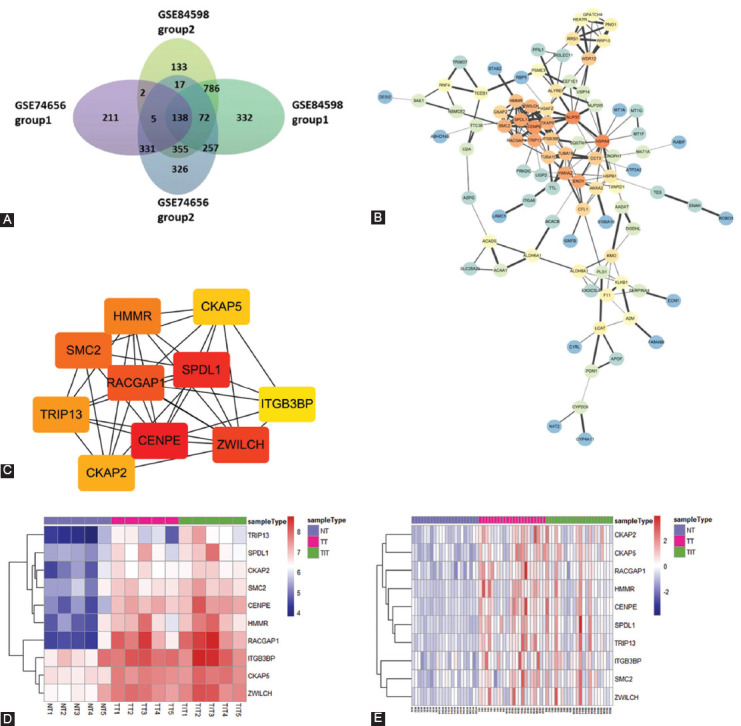
Differentially expressed genes (DEGs) identified in GSE74656 and GSE84598. (A) Venn diagram of the overlapping DEGs between GSE74656 and GSE84598. (B) Protein-protein interactions network of 138 SDEGs. Orange nodes have the highest degree while blue nodes have the lowest degree. The smaller the edge, the lower the combined score. (C) The top 10 hub genes screened using the Cytohubbo module in Cytoscape. Darker node color indicates a higher score. (D) Heat map of the top 10 hub genes expression in GSE74656. (E) Heat map of the top 10 hub genes expression in GSE84598.

Using R software “limma” package, we identified 1059 DEGs in group 1 (TT vs. NT) and 1484 DEGs in Group 2 (TIT vs. NT) among GSE74656. Meanwhile, in GSE84598, we found 4952 and 1153 DEGs in Group 1 and Group 2, respectively. However, there was no DEGs between TT and tumor invasion tissues (Group 3) (Table 1). The overlaps between GSE74656 and GSE84598 were illustrated in the Venn diagram (Figure 1A). Among the common DEGs of GSE74656 and GSE84598, a total of 138 SDEGs were differentially expressed both in Group 1 and Group 2 (Figure 1A).

**TABLE 1:**
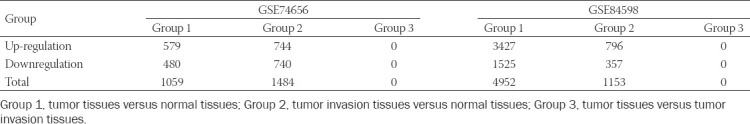
Number of DEGs in GSE74656 and GSE84598

### Functional annotation and PPI analysis of 138 SDEGs

As these 138 protein-coding genes might be associated with tumor invasion, they were inputted into the online tool KOBAS 3.0 for functional annotation. The results are shown in Table 2. KEGG pathway enrichment analysis found that these 138 SDEGs were mainly enriched in the “Metabolic pathways” pathway (P_adj_ = 8.21E-08) and involved in carboxylic acid metabolic process (P_adj_ = 0.0004). Furthermore, the PPI network was constructed using STRING and visualized with Cytoscape software. As shown in Figure 1B, some genes have higher degree and combined score in PPI network such as CENPE and SPDL1. Then, the top 10 hub genes were screened using the Cytohubbo module in Cytoscape for further analysis (Figure 1C). The top 10 hub genes were all upregulated in HCC TTs and TITs compared to NTs in GSE74656 (Figure 1D) and GSE84598 (Figure 1E).

**TABLE 2:**
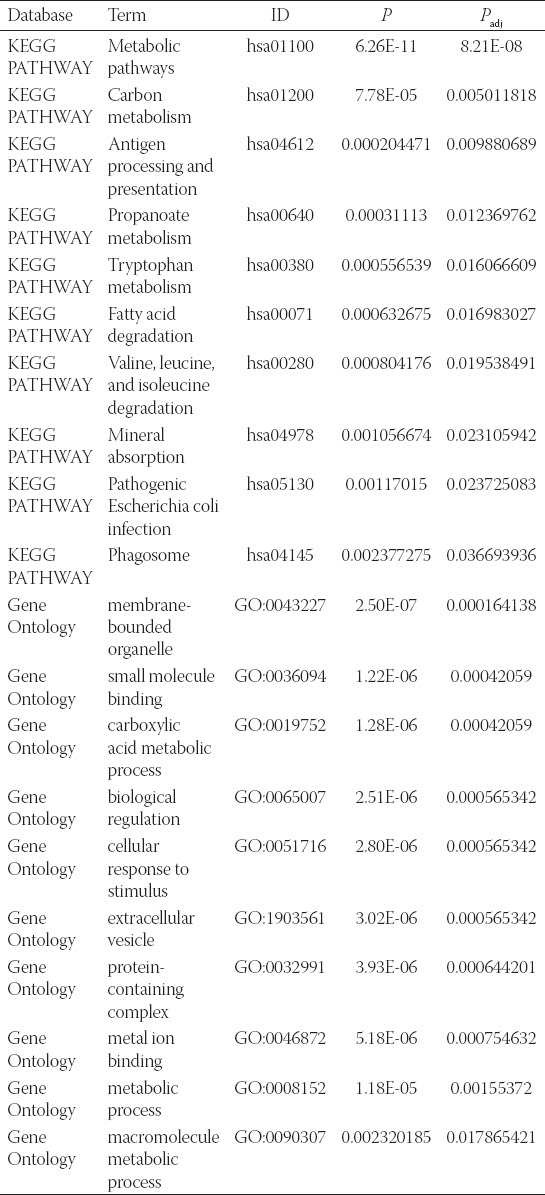
Top 10 significant pathway enrichments and functional annotations of 138 SDEGs

### Validating the expression of hub genes in TCGA and screening for prognostic hub genes

**FIGURE 2:**
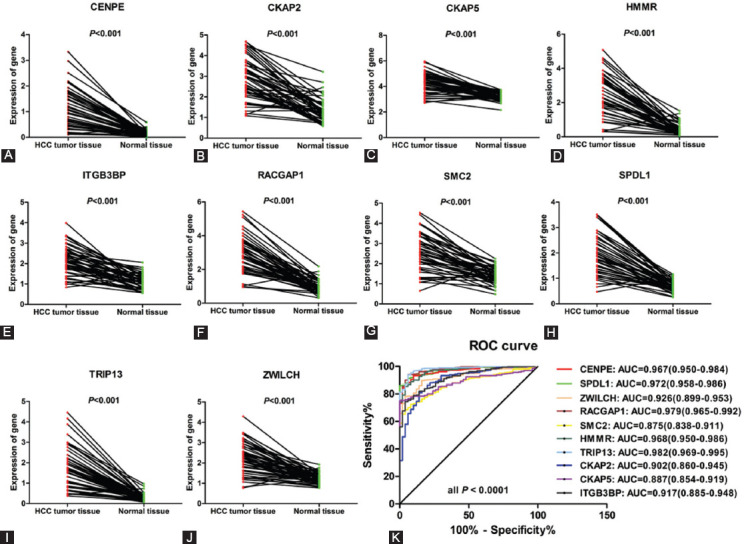
The expression level of hub genes of HCC patients in TCGA database. (A-J) Paired t-test showed that the 10 hub genes were upregulated in HCC tumor tissues. (K) ROC curve analysis showed that hub genes had good diagnostic efficacy for HCC.

We further validated the expression of hub genes in TCGA database. As shown in Figure 2A-J, all the 10 hub genes were upregulated in HCC TT compared with NT (*p* < 0.001). The ROC curves showed that the AUC of hub genes was >0.85, and the AUC of TRIP13 and RACGAP1 reached up to 0.982 and 0.979, respectively (Figure 2K), indicating that these 10 hub genes had good diagnostic efficacy for HCC.

**FIGURE 3:**
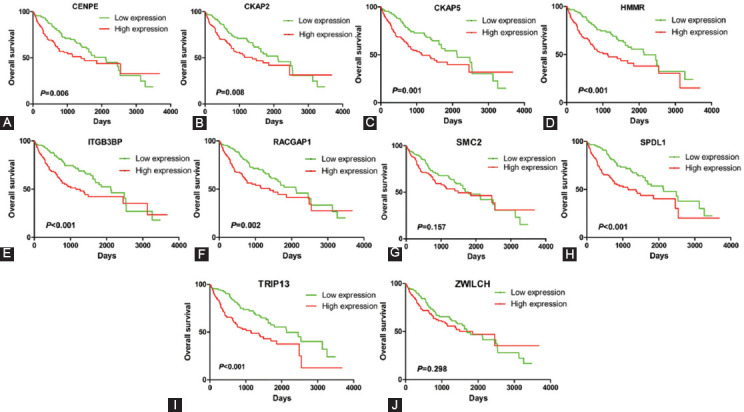
The relationship between hub gene expression and overall survival of HCC patients in TCGA database. The expression levels of 8 hub genes CENPE (A), CKAP2 (B), CKAP5 (C), HMMR (D), ITGB3BP (E), RACGAP1 (F), SPDL1 (H), and TRIP13 (I) are associated with overall survival of HCC patients.

**FIGURE 4:**
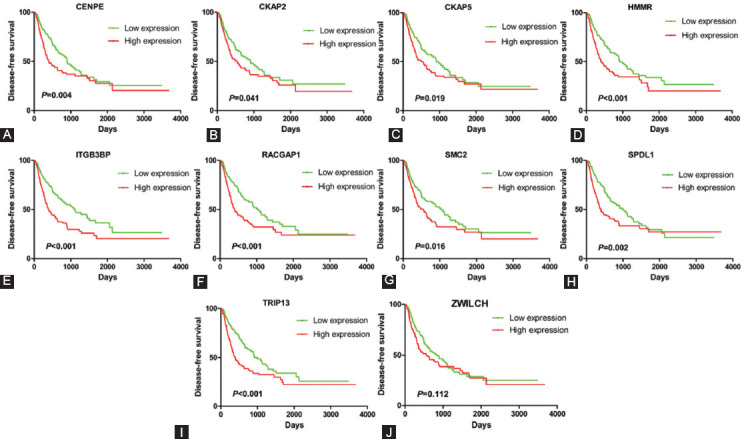
The relationship between hub gene expression and disease-free survival of HCC patients in TCGA database. The expression levels of 9 hub genes CENPE (A), CKAP2 (B), CKAP5 (C), HMMR (D), ITGB3BP (E), RACGAP1 (F), SMC2 (G), SPDL1 (H), and TRIP13(I) are associated with disease-free survival of HCC patients.

Then, we divided the patients into high-expression and low-expression group using the median of gene expression as the cutoff value, and analyzed the relationship between gene expression and prognosis in 371 HCC samples. As show in Figure 3, the expression level of 8 hub genes (CENPE, CKAP2, CKAP5, HMMR, ITGB3BP, RACGAP1, SPDL1, and TRIP13) was associated with OS of HCC patients. Similarly, the expression level of 9 hub genes (CENPE, CKAP2, CKAP5, HMMR, ITGB3BP, RACGAP1, SMC2, SPDL1, and TRIP13) was associated with disease-free survival (DFS) of HCC patients (Figure 4). Collectively, eight out of the 10 hub genes (CENPE, SPDL1, HMMR, RACGAP1, TRIP13, CKAP2, CKAP5, and ITGB3BP) were considered as prognostic hub genes.

### Prognostic hub genes are independent prognostic factors for HCC in TCGA database

**FIGURE 5:**
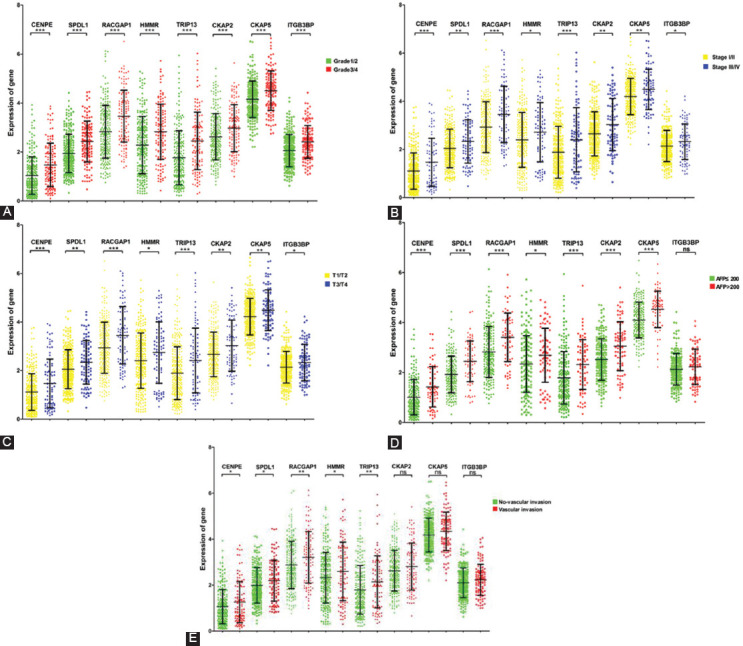
The expression levels of prognostic hub genes are related to some clinicopathological characteristics of HCC patients. (A) The expression of all prognostic hub genes is associated with histology grade of HCC. (B) The expression of all prognostic hub genes is associated with pathological stages of HCC. (C) The expression of all prognostic hub genes is associated with T stages of HCC. (D) The expression of CENPE, SPDL1, RACGAP1, HMMR, TRIP13, CKAP2, and CKAP5 is associated with AFP level of HCC patients. (E) The expression of CENPE, SPDL1, RACGAP1, HMMR, and TRIP13 is associated with vascular invasion of HCC. (*p < 0.05; **p < 0.01; ***p < 0.001; ns, not significant).

Analysis revealed that the expression of the eight prognostic hub genes was significantly associated with histology grade (Figure 5A), pathological stages (Figure 5B), and T stages (Figure 5C) in HCC patients (*p* < 0.05). The higher the gene expression, the higher the histology grade, pathological stages, and T stage in patients. Likewise, the expression of seven of the 8 prognostic hub genes (CENPE, SPDL1, RACGAP1, HMMR, TRIP13, CKAP2, and CKAP5) was significantly related to the AFP level in HCC (*p* < 0.05) (Figure 5D). Furthermore, the expression of five of the 8 prognostic hub genes (CENPE, SPDL1, RACGAP1, HMMR, and TRIP13) was significantly associated with the vascular invasion of HCC patients (*p* < 0.05) (Figure 5E). Multivariate cox regression analysis indicated that all the prognostic hub genes were independent prognostic factors for OS or DFS of HCC patients when adjusted by age, gender, histology grade, T stage, AFP level, and vascular invasion (Table 3).

**TABLE 3:**
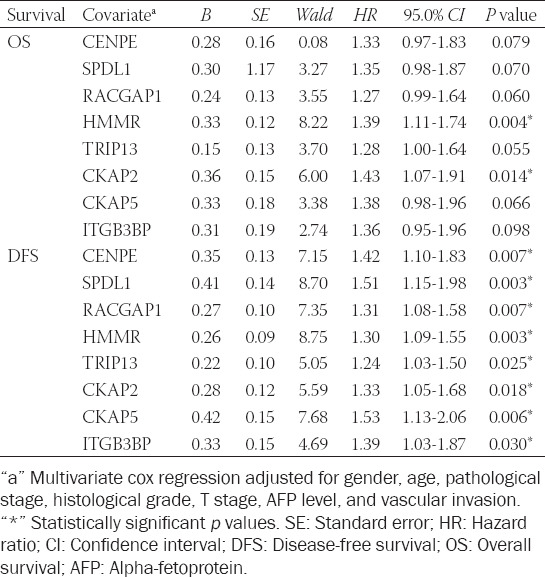
Prognostic value of hub genes was analyzed with adjustments for clinicopathological features of patients

### The 8-hub genes model has better predictive value for HCC survival

**FIGURE 6:**
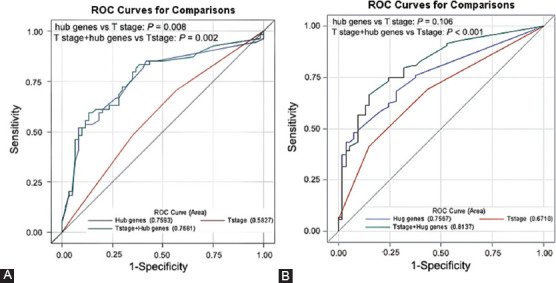
The predictive value of the 8-hub genes model for HCC survival. (A) The receiver operating characteristic (ROC) curves and area under the curves (AUCs) estimation for prediction of 1-year overall survival. (B) The ROC curves and AUCs estimation for predictive of 1-year disease-free survival.

To further evaluate the predictive value of the hub genes for HCC, we constructed a model with 8 hub genes in ROC curves analysis (Figure 6). As classification of 1-year OS, the AUC of the 8-hub genes model (AUC = 0.759) was significantly increased compared to the T stage (AUC = 0.583) (*p* = 0.008). After constructing the model with T stage and 8 prognostic hub genes, the AUC increased to 0.768, which significantly improved the predictive value of T stage (*p* = 0.002) (Figure 6A). For 1-year DFS, the AUC was significantly increased from 0.671 to 0.814 when adding the 8 hub genes to T stage as classifiers in the ROC curve (*p* < 0.001) (Figure 6B).

### Validating the protein expression levels of prognostic hub genes in The Human Protein Atlas

**FIGURE 7:**
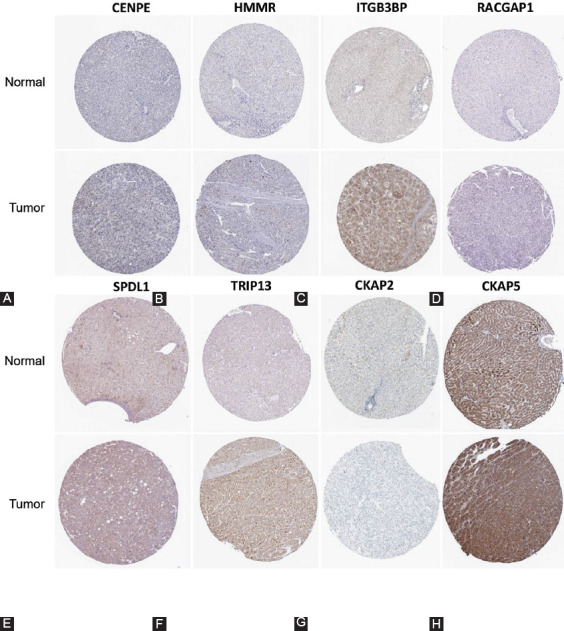
The protein expression levels of prognostic hub genes in The Human Protein Atlas. Then results of immunohistochemical staining of CENPE (A), HMMR (B), ITGB3BP (C), RACGAP1 (D), SPDL1 (E), TRIP13 (F), CKAP2 (G), CKAP5 (H).

Base on the predictive value of prognostic hub genes in HCC, we further verified their protein expression levels in HCC tissues according to the data of IHC downloaded from The Human Protein Atlas. The representative pictures are shown in Figure 7. We found that CENPE (0/7, *p* = 0.008), SPDL1 (4/6, *p* > 0.05), and CKAP2 (1/6, *p* = 0.048) were low stained only in a few HCC tissues but in all NT. These results were inconsistent with the results of mRNA. In addition, HMMR (1/6), RACGAP1 (1/6), and TRIP13 (2/7) were only detected in a few HCC tissues (*p* > 0.05). In particular, CKAP5 was stained in all tissues (three medium stained in three NT, two medium, and five high stained in seven HCC tissues, *p* > 0.05), and ITGB3BP was stained in all HCC tissues (four medium and two high stained in six HCC tissues) but not detected in NT (*p* = 0.012). The above results indicated that only the protein expression of ITGB3BP was upregulated in HCC tissues compared with NT. Therefore, we further evaluate the expression level of ITGB3BP in our cohort.

### ITGB3BP is upregulated and associated with lymph node metastasis in HCC

**FIGURE 8:**
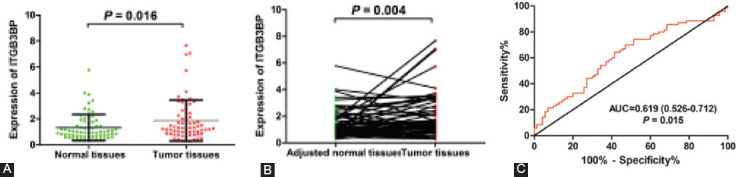
The expression level of ITGB3BP in HCC samples. (A) The mRNA expression level of ITGB3BP is up-regulated in HCC tissues. (B) Paired t-test of ITGB3BP expression in HCC tissues compared with para-carcinoma tissues. (C) ROC curve analysis of diagnostic efficacy of ITGB3BP expression for HCC.

We obtained 69 pairs of HCC tissues and their adjacent NT from the Affiliated Cancer Hospital of Guangxi Medical University. The results of qRT-PCR suggested that the mRNA level of ITGB3BP was upregulated in TT compared with NT (Figure 8A and B). The ROC curve analysis showed that ITGB3BP could distinguish TT from NT (AUC = 0.619, CI 0.525-0.713, *p* = 0.016) (Figure 8C). To further explore the clinical significance of ITGB3BP, we divided the patients into the high-expression group and the low-expression group with the median value of ITGB3BP expression (1.34) as the cutoff value. The results revealed that patients with high ITGB3BP expression were associated with lymph node metastasis that affecting the prognosis of HCC patients (*p* = 0.035). However, there was no significant correlation in gender, age, liver cirrhosis, tumor number, tumor size, microvascular invasion, hepatitis B virus infection, Child-Pugh classification grade, BCLC stage, and edmondson stage between two groups (Table 4).

**TABLE 4:**
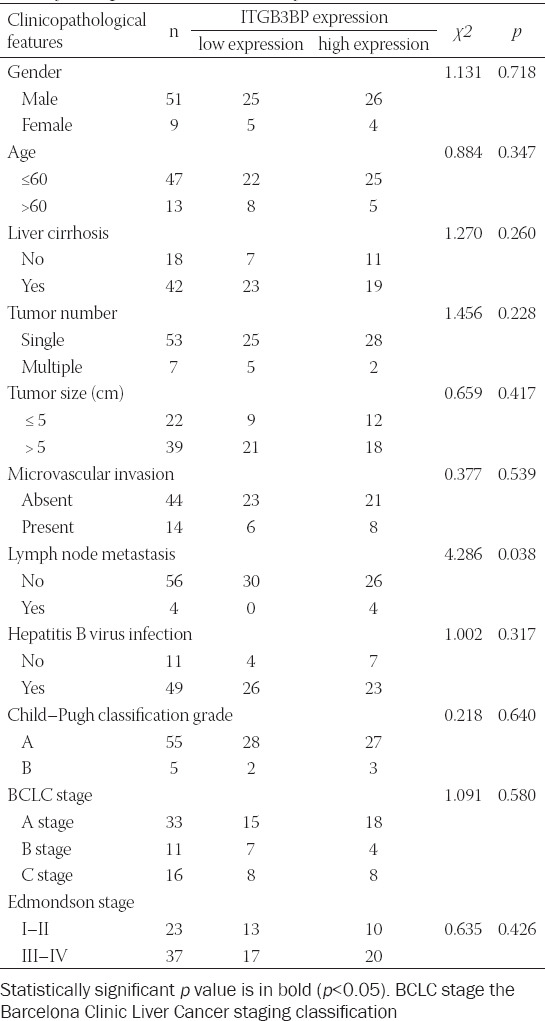
Association between ITGB3BP expression and clinicopathological features in HCC samples

## DISCUSSION

HCC is highly malignant and many patients develop recurrence and metastasis after systematic treatment, resulting in poor survival. Predicting the outcome and searching for prognostic biomarkers are important in the management of HCC patients after treatment. However, there are still few specific biomarkers used in the clinical treatment of HCC. Therefore, to reduce the mortality and improve the prognosis of HCC, screening of biomarkers for HCC is urgently needed. In this study, we screened 138 genes from GEO database and obtained the 10 most significant hub genes using the cytoHubba kit tool. Compared with NT, the expression of all hub genes in TT was significantly upregulated, of which eight hub genes (CENPE, SPDL1, HMMR, RACGAP1, TRIP13, CKAP2, CKAP5, and ITGB3BP) were identified as prognostic hub genes and independent prognostic factors for HCC. Further analysis revealed that the predictive performance of the model constructed by 8 hub genes for HCC survival was better than that of T stage, and can significantly improve the prediction value of the T stage.

In HCC patients from TCGA database, high expression of the eight prognostic hub genes was associated with poor prognosis. Besides, the expression levels of all prognostic hub genes were associated with histology grade, pathological stage and T stage in HCC patients, and the expression levels of some prognostic hub genes were associated with AFP level and vascular invasion. The histology grade, pathological stages, and T stages were found to be negatively correlated with the prognosis of HCC patients [15-17], which suggested that the expression levels of prognostic hub genes could distinguish between different histology grades, pathological stages, and T stages of HCC and predict the prognosis of HCC patients. Previous study has shown that the survival prognosis was significantly lower in patients with higher AFP level [18]. A positive correlation between AFP level and gene expression level was found in our research, indicating that the higher the gene expression level, the poorer the prognosis of the HCC patients, which was consistent with the results of survival analysis. Vascular invasion is known to be a predictor of early HCC recurrence [19] and is related to AFP, tumor size, and tumor number [20,21]. In the present study, the expression levels of upregulated genes were higher in HCC patients with vascular invasion compared to patients without vascular invasion. Multivariate cox regression analysis revealed that all prognostic hub genes are independent prognostic factors for HCC. The above results indicated that these genes are closely related to the clinical prognosis of HCC patients.

HCC has a high potential for recurrence and metastasis. Finding effective methods to evaluate the prognosis of HCC and guide the optimization of treatment strategies is an effective way to improve the survival time of patients. The American Joint Committee on Cancer tumor-node-metastasis (TNM) staging system is used to assess postoperative prognosis and predict postoperative survival, which is currently widely accepted and applied for HCC staging. However, this staging system still has some limitations [22,23]. In the present study, the AUC of the model constructed with 8 genes was larger than that of the T stage, indicating that the 8-hub genes model was of better predictive value for HCC survival than T staging system. Moreover, the combination of 8 prognostic hub genes and T stage significantly improved the predictive value of the model for HCC survival. Together, the 8-hub genes model may be able to more accurately predict the prognosis of HCC patients, especially when the 8 hub genes are combined with T staging system, which can better guide the post-operative treatment of HCC patients.

Of the eight prognostic hub genes we found, dysregulation of four including RACGAP1, TRIP13, CKAP2, and HMMR in HCC has been previously demonstrated to be associated with the progression and prognosis of HCC. RACGAP1 is a cytokinesis-regulatory protein. Overexpression of RACGAP1 promoted the proliferation of HCC cells by reducing the activation of Hippo and YAP pathways, increased cell division in collaboration with TPR, and was associated with shorter survival time of patients and high risk of early recurrence in patients after surgery [24,25]. As a tumor promoter, TRIP13 induced the proliferation, migration, invasion, metastasis, and inhibited cell apoptosis of HCC by acting on the TGF-binding 1/smad3 or AKT/mTOR signaling pathway. Patients with high TRIP13 expression had significantly poor survival [26-28]. Similarly, the CKAP2 was associated with early and widespread recurrence in patients with HCC after surgery. Patients with higher CKAP2 expression had shorter recurrence free survival time [29], which is consistent with our findings. HMMR promotes the proliferation of HCC cells *in vitro* by activating G1/S and G2/M checkpoint transformation, and knockdown of HMMR suppresses HCC tumor growth in nude mice [30]. Other studies have found that CENPE [31], SPDL1 [32], and CKAP5 [33] were also associated with the prognosis of HCC through bioinformatics analysis. These findings suggest that the hub genes may play important roles in HCC development and progress.

ITGB3BP, also known as CENPR, NRIF3, and TAP20, is located in the nucleus and is a nuclear receptor coactivator that exhibits a distinct receptor specificity [34]. ITGB3BP had been demonstrated to be involved in the progression of multiple cancers, including HCC. Dangsheng Li *et al*. found that breast cancer cells contain a novel “death switch,” which may be specifically triggered by ITGB3BP, selectively inducing rapid apoptosis of breast cancer cells [35]. Furthermore, ITGB3BP and DIF-1 complexes selectively control cell apoptosis of breast cancer by regulating FASTKD2 [36]. Overexpression of ITGB3BP was demonstrated to decrease the proportion of side population (SP) cells which were harboring malignant phenotypes in ovarian cancer [37]. Moreover, in the skin cancer, ITGB3BP was documented to play a dual role: It acts as a tumor suppressor in the early stages of tumorigenesis while acting as a promoter in the progression of cancer [38]. Changzhou Cai *et al*. found that the high expression of ITGB3BP was associated with poor prognosis in HCC patients and verified the expression of ITGB3BP at the cellular level. However, they did not find high expression of ITGB3BP in some HCC cell lines due to the heterogeneity of the tumor or other reasons [39]. In this study, we selected samples different from Changzhou Cai *et al*. to verify the expression level of ITGB3BP in Human Protein Atlas and our cohort, and then we analyzed the relationship between the expression level and the clinical characteristics of HCC patients. It was confirmed that ITGB3BP was upregulated in HCC, and the high expression of ITGB3BP was significantly correlated with lymph node metastasis.

It is worth noting that this study has certain limitations. First, the study used a relatively a few samples for preliminary screening of genes. However, we verified the genes in the TCGA database, which contains a larger sample size. Second, no experimental study *in vitro* or *in vivo* was conducted to investigate the role and mechanism of some of the genes in HCC. However, studies have shown that some of them play an important role in the progression of other tumors, such as breast cancer, gastric cancer, and lung adenocarcinoma [35,40,41]. Therefore, we need to further verify the role of these genes in HCC in future studies.

## CONCLUSION

In summary, the present study uses bioinformatic analysis to analyze DEGs that might be associated with invasion in HCC and identified 8 hub genes were associated with prognosis of HCC patients. Importantly, we constructed a 8-hub genes model that has better prediction performance than the T staging system in prognosis prediction of HCC patients. One of the prognostic hub genes, ITGB3BP, was confirmed that upregulated in HCC, and the high expression of ITGB3BP was significantly correlated with lymph node metastasis. It provides important clues for prognosis prediction and precise treatment of HCC.
